# Forgetfulness, Etc.

**Published:** 1881-07-20

**Authors:** 


### *"FORGETFULNESS, CARELESSNESS, PROCRASTINATION."*

"A hearty confession is good for the soul." A subscriber from North Carolina has relieved his conscience by remitting his dues after long indulgence on our part. He thus writes:

DR. R. C. WORD, Atlanta, Georgia:

*Dear Sir*—You will please be sure *not* to publish the receipt of this *in the Journal;* on the contrary, be *very private* about acknowledging it; and that you do it as much in the dark as possible, I enclose an envelope. I am ashamed of my long neglect of your claims upon me, which you may attribute to a commingling of forgetfulness, carelessness and procrastination. However, I send you money to pay for back dues. The RECORD, like wine, grows better as it gets older, and I congratulate you upon its high position in the medical world.

Yours truly, B. T. M.

This tardy confession of our friend restores him to our confidence and shows him to be a true man at heart; and yet this habit of procrastination and neglect, though repented of at last, does not wholly wipe out the injury and loss inflicted upon the journalist who is forced to meet the cash demands of the printer under serious perplexities and disadvantages. This course is not meant to rebuke the friend who now so nobly confesses, but to give a much needed hint to the many yet in arrears who continue to disregard our reminders of the fact. Yes, "*carelessness*, postponement, forgetfulness," are the breakers upon which so many good and noble literary enterprises in the South have been hopelessly wrecked. W.

